# Simultaneous label-free autofluorescence-multiharmonic microscopy and beyond

**DOI:** 10.1063/1.5098349

**Published:** 2019-10-01

**Authors:** Stephen A. Boppart, Sixian You, Lianhuang Li, Jianxin Chen, Haohua Tu

**Affiliations:** 1 Biophotonics Imaging Laboratory, Beckman Institute for Advanced Science and Technology, University of Illinois at Urbana-Champaign, Urbana, Illinois 61801, USA; 2 Key Laboratory of OptoElectronic Science and Technology for Medicine of Ministry of Education, Fujian Provincial Key Laboratory of Photonics Technology, Fujian Normal University, Fuzhou 350007, China

## Abstract

Without sophisticated data inversion algorithms, nonlinear optical microscopy can acquire images at subcellular resolution and relatively large depth, with plausible endogenous contrasts indicative of authentic biological and pathological states. Although independent contrasts have been derived by sequentially imaging the same sample plane or volume under different and often optimized excitation conditions, new laser source engineering with inputs from key biomolecules surprisingly enable real-time simultaneous acquisition of multiple endogenous molecular contrasts to segment a rich set of cellular and extracellular components. Since this development allows simple single-beam single-shot excitation and simultaneous multicontrast epidirected signal detection, the resulting platform avoids perturbative sample pretreatments such as fluorescent labeling, mechanical sectioning, scarce or interdependent contrast generation, constraints to the sample or imaging geometry, and intraimaging motion artifacts that have limited *in vivo* nonlinear optical molecular imaging.

## I. LABELFREE MOLECULAR NONLINEAR OPTICAL MICROSCOPY WITH COUPLED LASER SOURCE ENGINEERING

Nonlinear optical microscopy is a well-established biological imaging technology that complements wide-field phase-contrast/fluorescence microscopy and laser-scanning confocal microscopy, with unique capabilities to molecularly and orthogonally segment cellular and extracellular components in untreated unlabeled tissue, even plausibly in live animals and humans.^1,2^ Therefore, we largely limit the scope of this Perspective to label-free molecular nonlinear optical microscopy, which we treat as a prerequisite to directly translate optical imaging-based knowledge from cell and animal biology to human medicine.^3^ Diverse and well-known nonlinear optical processes (modalities) of endogenous biomolecules have uniquely enabled this approach. These include autofluorescence (AF) such as two-photon or three-photon excited AF (2PAF or 3PAF),^3^ second-harmonic generation (SHG),^4^ third-harmonic generation (THG),^5^ and coherent Raman scattering (CRS) in the form of coherent anti-Stokes Raman scattering (CARS) or stimulated Raman scattering (SRS).^6^ It is generally agreed that maximal integration of these complementary modalities is needed to realize broadly applicable labelfree nonlinear optical imaging with molecular specificity.^7^

**FIG. 1.**
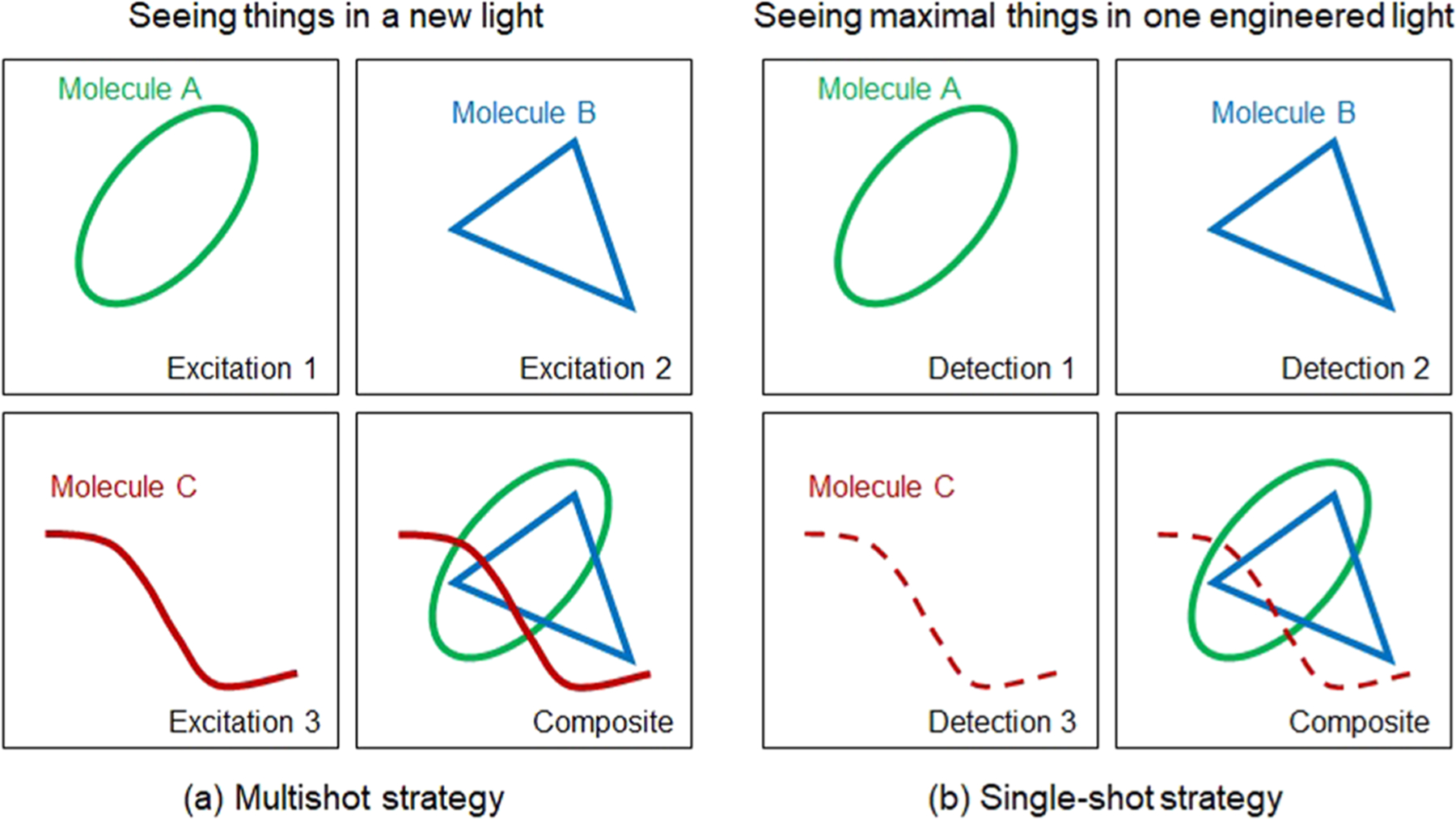
(a) Multishot strategy for “seeing (different) things in a new light.” (b) Single-shot strategy for “seeing maximal things in one engineered light.” Spatial distribution of molecule C may be visualized and differentiated against background molecules (A and B) by the multishot strategy, but not by the single-shot strategy.

For a given biomolecule of interest, labelfree nonlinear optical imaging seeks an optimal and often exotic laser excitation to specifically visualize this molecule, rather than to label it with an extrinsic agent (fluorescent dye/antibody, Raman/absorption probe, etc.) and to visualize it under a fixed and readily available excitation condition. This is why the corresponding microscopy development has always been tightly coupled with ultrafast laser source engineering.^8^ To broadly impact bioscience and medicine, two competing strategies of laser source engineering with intrinsic trade-offs have been employed (Fig. 1). One strategy, termed as “seeing (different) things in a new light,” invokes different excitation conditions (shots) and plausibly different signal detection conditions along one channel (by one photodetector) to sequentially visualize the distribution of different biomolecules [Fig. 1(a)]. Thus, the number of shots at one image pixel scales with the number of biomolecules intended to image, causing an intraimaging motion artifact for dynamic samples to co-register different molecular contrasts if molecularly specific images [Fig. 1(a), upper left, upper right, and lower left] are acquired frame-by-frame.^9^ Although laser source engineering with fast tuning speeds may enable sequential imaging of different biomolecules at each pixel to reconstruct the molecularly specific images [Fig. 1(a), lower right] without this artifact, the technique introduces additional complexity and the elongated pixel dwell time may raise the risk of photodamage in *in vivo* imaging.

An alternative strategy for laser source engineering, termed “seeing maximal things in one engineered light,” has also been explored to image multiple biomolecules in one shot, i.e., under one excitation condition but with different signal detection conditions and channels [Fig. 1(b)].^1,3,7^ In comparison to its multishot counterpart, this single-shot strategy simultaneously would avoid the photodamage risk and intraimaging motion artifact and thus improve photon economics. However, these advantages are offset by a lack of orthogonal (independent) molecular contrasts that can be either spectrally resolved or spatially segmented. It is questionable whether one “smart” choice of a fixed excitation condition could simultaneously image a comprehensive set of biomolecules. The single-shot strategy would certainly miss some biomolecules that can only be optimally excited and imaged by the multishot strategy [Fig. 1(b), lower left], and would seem inadequate to image chemically complex tissue. This limiting factor is worsened by the conflict between CRS modalities and less molecular-specific multiphoton modalities (i.e., 2PAF, 3PAF, SHG, and THG) that employ different and often incompatible conditions. We thus treat the CRS modalities and multiphoton modalities as two distinct categories of nonlinear optical microscopy. Even within the scope of the multiphoton modalities, it is technically difficult to generate signals with comparable signal-to-noise (SNR) ratios across 2PAF, 3PAF, SHG, and THG by one excitation condition, which depend not only on excitation parameters but also on the multimodal detection schemes and associated photodetectors. Within the smaller scope of fluorescence imaging (2PAF and 3PAF), the single-shot strategy does not have the capability of its multishot counterpart to unmix the spectrally overlapped fluorescence from two fluorophores, and will rely more on spectral separation at the point of image acquisition.

In our opinion, the aforementioned multishot strategy is useful to visualize a biomolecule of interest in *in vitro* cell or tissue cultures, thin *ex vivo* tissue sections, or small living organisms but is ill suited to image the same molecule in the molecular context of dynamic and *in vivo* animal or human conditions. This intrinsic obstacle cancels the ultimate advantage of labelfree nonlinear optical microscopy in *in vivo* samples across different species since under the former condition the same molecule could be fluorescently labeled and alternatively imaged by more cost-effective laser-scanning or wide-field fluorescence microscopy, with likely better sensitivity and molecular specificity. Thus, this Perspective will advocate the transition of laser source engineering from the multishot strategy toward the single-shot strategy (Fig. 1), and discuss and envision how the lack of orthogonal molecular contrasts can be mitigated or overcome. Optical physicists and laser engineers are therefore required to understand and identify key endogenous biomolecules pertinent to nonlinear optical microscopy, and tailor their laser source engineering to visualize them in one shot. This biomolecule-targeted laser source engineering is rather different from conventional laser source engineering with no input from biomolecules, which has often evolved toward more flexibility, versatility, complexity, and cost to inadvertently limit the widespread use of nonlinear optical microscopy.

## II. LASER-MICROSCOPE PLATFORMS AND *IN VIVO* APPLICABILITY

For *in vivo* samples, the applicability of a laser-microscope platform for labelfree molecular nonlinear optical microscopy can be quantitatively evaluated by five elements of binary choice in decreasing scale of importance (from 5 to 1) (Table I). First, as discussed above, it is critical to choose the single-shot strategy over the multishot strategy (Fig. 1). Second, CRS modality should be included whenever possible due to its high molecular specificity. Third, multiple imaging modalities^7^ and independent spectroscopic detection/excitation (orthogonal content) is preferred over their singular and nonspectroscopic counterparts to gain high molecular content and improve photon economics.^11^ Fourth, backward (reflective, epi) signal detection should be chosen over its forward counterpart to expand imaging to thick intact *ex vivo* tissue, or to live animals or humans.^1,11^ Finally (fifth), single-beam (or single-pulse) excitation is preferred over its multibeam counterpart to reduce the complexity of the excitation laser source.^12^ With the establishment of these preselected 5-element metrics, termed a “configuration vector,” the *in vivo* applicability of a specific laser-microscope platform can be scored and weighed in against the associated limiting factor(s) (Table I).^3,9,10,13–15^ There exists no correspondence between the quantifiable applicability and the number of integrated modalities (Table I), which has been a frequent misconception to build a multimodal nonlinear optical microscopy.

**FIG. 2.**
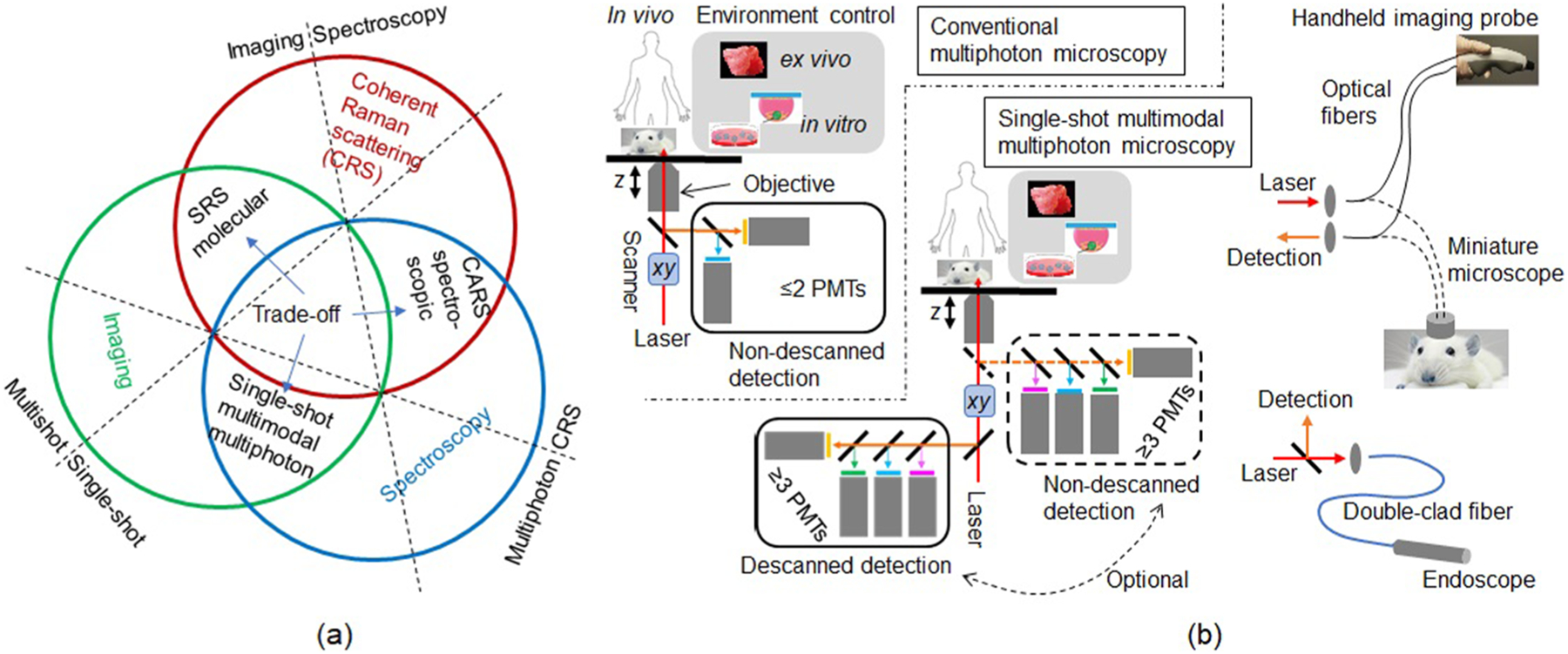
(a) Tradeoff in labelfree nonlinear optical imaging among SRS molecular, CARS spectroscopic, and single-shot multimodal multiphoton single-shot microscopy that arises from the imaging-spectroscopy trade-off, single-shot vs multishot trade-off, and multiphoton-CRS tradeoff. (b) Comparison of optical schematics between conventional multiphoton microscopy and single-shot multimodal multiphoton microscopy (shown as inverted geometry but can be modified to attain upright geometry); the later resembles a commercial benchtop laser-scanning confocal microscope and can be implemented as fiber-coupled devices (handheld imaging probe, head-mount miniature microscope, and endoscope) to flexibly access different samples and tissue sites.

The direct comparison between the platform conducive to fast but sequential visualization of one biomolecule (SRS molecular)^13^ and slow but simultaneous visualization of multiple biomolecules (CARS spectroscopic)^14^ highlights the intrinsic imaging-spectroscopy trade-off [Table I, Fig. 2(a)]. The imaging gains high molecular specify for a targeted biomolecule at the cost of its molecular context that can only be acquired spectroscopically. The radical way to avoid this trade-off is to reject CRS and instead derive molecular specificity exclusively from the multiphoton modalities (2PAF, 3PAF, SHG, and THG), resulting in single-shot multimodal (≥3 modalities) multiphoton microscopy^3,10^ to complement the SRS molecular and CARS spectroscopic microscopy [Fig. 2(a)]. This picture introduces a new CRS-multiphoton trade-off in addition to the multishot vs single-shot trade-off (Fig. 1) and the imaging-spectroscopy trade-off [Fig. 2(a)]. To balance these trade-offs, other platforms have adopted less radical ways (incorporated hyperspectral CRS imaging) to attain adaptive (programmable) multishot operation and simple single-beam multimodal integration (Table I).^9,15^ These and numerous other platforms have obtained a variety of hybrid CRS molecular, CARS spectroscopic, and single-shot multimodal multiphoton microscopy [Fig. 2(a)] but have not increased the *in vivo* applicability score over the single-shot multimodal multiphoton microscopy (Table I), highlighting the incompatibility between CRS and multiphoton imaging modalities under the combined single-shot single-beam excitation and epidetection condition.

We therefore focus on the single-shot multimodal multiphoton microscopy that has high *in vivo* applicability. It should be noted that none of the binary choices in its 5-element configuration vector is unprecedented (Table I). For example, the epidetection of typically forward-directed SHG and THG signals is somewhat unconventional but has been demonstrated for thick/scattering tissue due to tissue backscattering.^1,16^ Strong THG signal can be acquired from thin/transparent specimens.^17^ As another example, the multichannel/color detection of multimodal episignals by an array (> 2 elements) of nondescanned photomultipliers (PMTs) would increasingly reject the multiply scattered signals collected by the photodetectors further away from the sample^11^ and would cancel one distinct advantage of multiphoton microscopy over confocal microscopy [Fig. 2(b)].^18^ However, the multiply scattered signals can be minimized in comparison to singly scattered signals by proper selection of the microscope objective,^19^ permitting the construction of the single-shot multimodal multiphoton microscopy based on a commercial benchtop laser-scanning confocal microscope using either descanned or nondescanned detection.^11^

The resemblance of the single-shot multimodal multiphoton microscopy to commercial laser-scanning confocal microscopy in the optical excitation and detection schematic may be beneficial for its potential widespread use via an optical fiber interface [Fig. 2(b)]. For this to happen, however, it is important to compare different forms of single-shot multimodal multiphoton microscopy under the constraint of one common configuration vector (Table I). It is possible to maximize the orthogonal molecular contrasts (to overcome the limiting factor of the single-shot strategy in Table I) by unique biomolecule-targeted laser source engineering, as shown below.

## III. BIOMOLECULE-TARGETED LASER SOURCE ENGINEERING FOR LIVE-TISSUE IMAGING

**FIG. 3.**
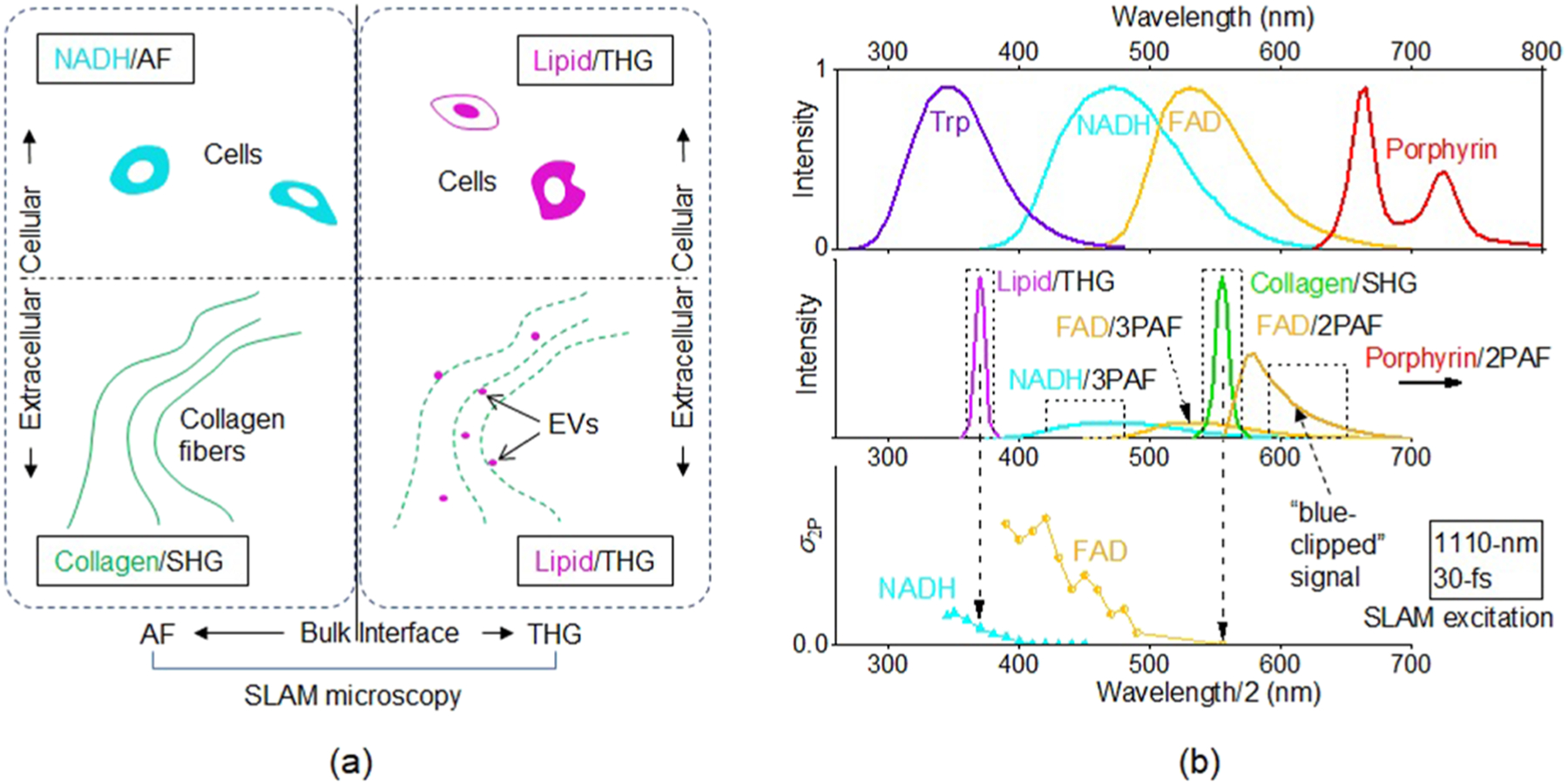
(a) Ternary tissue composition of NADH, collagen, and lipid targeted by laser source engineering of single-shot multimodal multiphoton microscopy to segment important cellular and extracellular components. (b) Top panel: emission spectra of popular endogenous fluorophores. Middle panel: obtainable signals from SLAM microscopy with spectrally resolved detection channels (dashed boxes). Bottom panel: spectra of two-photon absorption cross section (*σ*_2P_) of NADH and FAD [Reproduced with permission from Huang *et al.*, Biophys J. **82**(5), 2811 (2002). Copyright 2002 The Biophysical Society, published by Elsevier], in which a “smart” excitation condition places the SHG signal at the red edge of the FAD *σ*_2P_ spectrum to generate a “blue-clipped” FAD/2PAF signal.

Live-cell imaging in the unperturbed tissue environment calls for simultaneous imaging of general cellular and extracellular biomolecules to distinguish the cells from their surrounding matrix. Extensive research by multiphoton microscopy on diverse biological samples has yielded one well-accepted (if not optimal) combination of these biomolecules. On one hand, imaging of the endogenous cytoplasmic fluorophores of reduced nicotinamide adenine dinucleotide (NADH), and to a lesser extent, flavin adenine dinucleotide (FAD), by AF (mainly 2PAF) have structurally identified a broad variety of cells and functionally revealed their free or bound state via intensity and lifetime measurements.^20,21^ On the other hand, imaging of fiber-shaped collagen by SHG highlights the structure of the surrounding extracellular matrix, with plausible diagnostic importance.^22^ While this combination is useful, highly complementary imaging of optical heterogeneity by THG can independently identify the cells,^23^ as well as extracellular vesicles (EVs) that introduce function to the extracellular matrix [Fig. 3(a)].^24–26^ In contrast to AF/SHG imaging that provides bulk molecular distribution, THG imaging can reveal the interfacial/bilayer distribution of the membrane lipid,^3^ as well as protein-water interfaces (elastin, myelin, etc.) and lipid bodies (e.g., lipid vacuoles/droplets).^5,16^ We will later refer the membrane lipid and lipid bodies generally as “lipid.” The resulting picture presents a synergistic combination of 0D (EV), 1D (collagen), 2D (lipid-revealed membrane), and 3D (NADH-revealed cytoplasm) molecular structures to delineate both cellular and extracellular components [Fig. 3(a)]. Thus, specific laser source engineering is needed to simultaneously image these biomolecules by multiphoton microscopy.

Under the constraint of the configuration vector specific to single-shot multimodal multiphoton microscopy (Table I), three representative platforms have emerged to image these biomolecules. One dual-modal (2PAF-SHG) platform performs single-shot imaging on NADH at ~740 excitation,^3^ while the other dual-modal (THG-SHG) platform conducts imaging of lipid at ~1230 nm (Table II).^7,23^ Effort has been taken to collect autofluorescence signals in the latter platform but are found to be weak and uninformative in comparison to the harmonics signals.^27^ As to the former, the effort to integrate THG has been hindered by specific UV optics (e.g., microscope objective) to collect the lipid episignal under 250 nm. Another effort has carried out simultaneous 3PAF-2PAF imaging of tryptophan (Trp) and NADH to possibly improve the identification of specific cells (Table II),^28^ such as leukocytes.^29^ The spectrally well-resolved emission of Trp and NADH enables simple detection with no significant bleed-through [Fig. 3(b), top panel]. However, in contrast to the cellular imaging by NADH and lipid based on independent multiphoton processes, the cellular imaging by Trp and NADH may be interrelated due to Förster resonance energy transfer.^28^

In short, the above two highly complementary but hitherto incompatible platforms have been limited by the inability to image both NADH and lipid in one shot, i.e., to comprehensively visualize cells *in vivo* in a collagen-revealed extracellular matrix (Table II). Recent commercial development of a single-box widely tunable (700–1300 nm) optical parametric oscillator (OPO) can replace the two different lasers of the two platforms (Table II) to accommodate all imaging modalities. However, this full integration will not eliminate the possibility that a cell is invisible by the fixed-wavelength shot optimized for NADH (or lipid), but would otherwise be visible by the fixed-wavelength shot optimized for lipid (or NADH). Because NADH, collagen, and lipid have been, respectively, imaged at far apart “optimal” wavelength ranges of 700–760 nm (2PAF),^2^ 800–900 nm (SHG),^4^ and 1180–1350 nm (THG),^5^ respectively, it has been challenging to simultaneously image all of them in one fixed-wavelength shot with high molecular specificity and balanced SNRs, without slowing imaging speed or increasing the potential for photodamage. The reported Simultaneous Labelfree Autofluorescence-Multiharmonic (SLAM) microscopy has aimed to overcome this challenge.^10^ The key concept is to place the excitation wavelength at ~1110 nm amenable to both THG and SHG epidetected imaging,^30,31^ engineer the duty-cycle of typical lasers for multiphoton microscopy (~10^−5^) to attain a much lower value <10^−6^, and image lipid, NADH, collagen, and non-NADH yellow-red autofluorescent species (YRAFs) such as FAD by spectrally separated THG, 3PAF, SHG, and 2PAF episignals, respectively [Fig. 3(b), middle and bottom panels] (Table II).

**FIG. 4.**
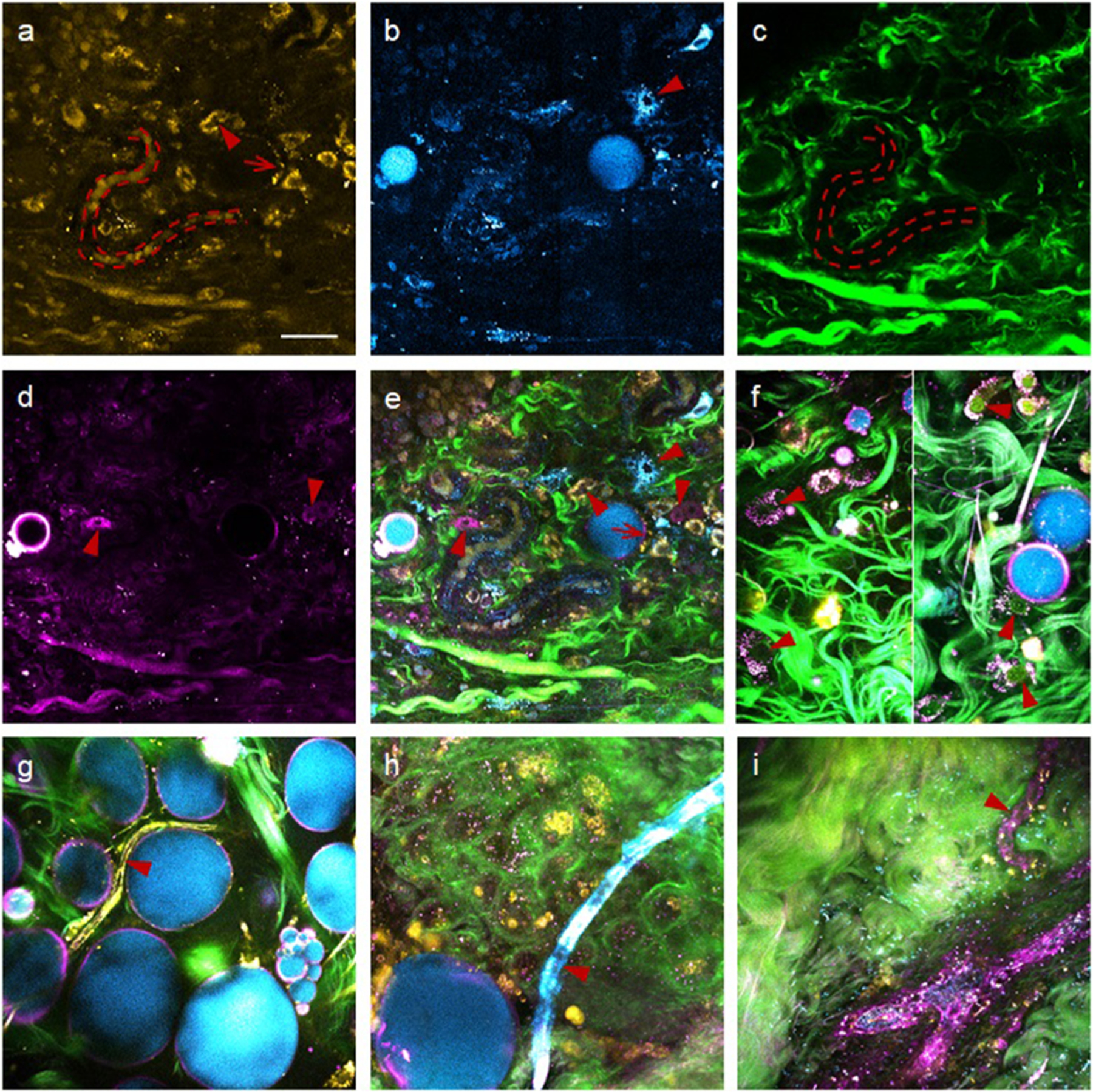
Imaging rat mammary and human breast specimens by SLAM microscopy. (a)-(d): 2PAF (yellow), 3PAF (cyan), SHG (green), and THG (magenta) images of an *in vivo* rat tumor microenvironment that highlight stromal cells (arrowheads), erythrocytes (broken curves), and an adipocyte (arrow). (e) Composite image of (a)-(d) with optically phenotyped cells of different colors (arrowheads). (f) Mitochon-drialike organelles in *ex vivo* human normal microenvironment (left panel, arrowheads) validated by labeling cell nuclei with acridine orange (right panel, arrowheads). (g)-(i) Capillary vessels in *ex vivo* human normal [(g) and (h)] and tumor (i) microenvironments that reveal different endothelial cells (arrowheads). Scale bar: 40 *μ*m (applicable to all fields of view).

The visualization of NADH by shifting the excitation wavelength from 740 nm (2PAF) to 1110 nm (3PAF) resembles the visualization of Trp by shifting the excitation wavelength from 590 nm (2PAF) to 740 nm (3PAF) (Table II).^28,29^ Regardless of the excitation wavelengths, the detection window of 420–480 nm is highly specific to NADH for a cell identifiable by NADH fluorescence, with negligible contamination from flavin adenine dinucleotide (FAD) and Trp [Fig. 3(b), top panel]. However, it has remained unknown whether this molecularly specific imaging can be rapidly and safely conducted at physiological NADH concentrations by 3PAF. In Figs. 4(a)–4(e), we have demonstrated continuous (>2 h) and safe (14 mW average power on sample) NADH/3PAF imaging in living rats at a relatively fast scanning/imaging speed (~0.5 *μ*m pixel size with ~10 *μ*s dwell time, or 500 × 500 pixelated frame with 250 × 250 *μ*m^2^ standard field-of-view at a frame rate of 0.5 Hz), both of which are comparable to typical NADH/2PAF imaging.^32^ Some NADH/3PAF-highlighted cells in the unperturbed *in vivo* microenvironment of a rat mammary tumor model can be structurally distinguished (image segmented) from lipid vacuoles that also fluoresce in this detection window [Fig. 4(b), arrowhead].^33^ The complete picture of this tumor microenvironment allows classification of all observed cells into diverse optical phenotypes (colors), including cyan-/magenta-/yellow-colored stromal cells of plausible immune origin [Fig. 4(e), arrowheads], a yellow-colored adipocyte associated with a cyan-colored lipid vacuole [Fig. 4(e), arrow], and yellow-colored erythrocytes flowing in a blood capillary [Fig. 4(a), broken curves]. Thus, YRAFs add to the ternary tissue composition of NADH, collagen, and lipid [Fig. 3(a)] the fourth orthogonal molecular contrast (yellow). It should be noted that Soret fluorescence from hemoglobin^34^ is hardly visible in the 3PAF channel to reveal these erythrocytes [Figs. 4(b) and 4(e)].

This balanced multicolor imaging and cell phenotyping is currently not available from multiphoton microscopy using commercial/conventional lasers (Table II) and the widely tunable OPO, all of which operate at a pulse repetition rate *f* of ~80 MHz and a pulse width *τ* of ~150 fs, or a duty-cycle *fτ* of ~10^−5^. These parameters constitute the most accessible condition from solid-state femtosecond lasers, but are not necessarily optimal for *in vivo* imaging. Because multiphoton-excited signals scale with *P*^*n*^/(*fτ*)^*n*−1^, where *P* is average power on the sample and *n* is the order of multiphoton excitation (*n* = 2 for 2PAF/SHG and *n* = 3 for 3PAF/THG), the same 3PAF/THG signals in Fig. 4(a) or Fig. 4(b) would require *P* > 140 mW if the OPO is tuned to the same excitation wavelength (1110 nm), which would likely cause photodamage to the sample.^35^ Lowering *P* to a safe level would not only slow down the imaging speed, but also produce attenuated 3PAF/THG signals that may then be obscured by less attenuated 2PAF/SHG signals according to the *P*^*n*^/(*fτ*)^*n*−1^ dependence. Alternatively, without lowering the duty-cycle, the orthogonal molecular contrasts of NADH, lipid, collagen, and YRAFs in Fig. 4 may be sequentially obtained using multiple shots (excitation wavelengths) by tuning the OPO or combining the two conventional lasers in Table II. However, this would eliminate the advantage of single-shot strategy in *in vivo* applications. It is then clear that the laser source engineering targeting these biomolecules with low-duty-cycle ~1110 nm pulses unavailable from commercial or previously reported lasers is necessary to enable this balanced multicolor imaging and cell phenotyping.^10^ Thus, SLAM microscopy should not be simply treated as a specific case of (multi-shot-natured) multimodal multiphoton microscopy that integrates numerous imaging modalities.^9^

The identification of orthogonally colored cells [Fig. 4(e), arrowheads] indicates that neither of THG (magenta color) and cellular NADH autofluorescence (cyan color), imaged separately by the two non-SLAM platforms in Table II, can serve as a universal signal to recognize cells in tissue. The cyan- or yellow-colored cells free of magenta color (THG) may arise from diverse chemical subtypes and/or structures of the membrane lipid that do not produce strong THG signals. Thus, the THG contrast that identifies specific cells [Fig. 4(d)] or subcellular organelles [Fig. 4(f)] in tissue contains both functional and structural information of these cells, and therefore parallels the fluorescence contrasts based on NADH/2PAF (cyan) and YRAFs/2PAF (yellow). In contrast to these rat cells that were identified *in vivo*, similarly colored subtypes of human endothelial cells forming different capillary vessels in freshly dissected mammary tissue were identified *ex vivo* [Figs. 4(g)–4(i), arrowheads]. While these specific rat and human cells are chosen to demonstrate the orthogonality among NADH/2PAF, YRAFs/2PAF, and lipid/THG (Fig. 4), a wide variety of cell types including epithelial cells in different organs have exhibited a mixture of cyan, yellow, and magenta colors with distinct morphologies, and can be correlated across species between *in vivo* (or intravital) rat mammary tissue and fresh *ex vivo* human breast tissue.^36^ For biomarker discovery, it is useful to molecularly profile or phenotype different cells in live tissue as well as subcellular organelles [Fig. 4(f)] and EVs that have been released into the stroma,^25^ i.e., to perform slidefree virtual histochemistry.^36,37^

As to the extracellular matrix, various vessels can be structurally and functionally assessed via their structural collagen/SHG and elastin/2PAF indirectly, and via constituent endothelial cells directly [Figs. 4(g)–4(i)]. Also, muscle, nerve, and adipose tissues can be first identified by their distinct SHG-visible myosin, THG-visible myelin, and THG-visible lipid-vacuoles (molecular contrasts of extracellular matrix, Table II), respectively, and their function can be subsequently profiled using the three colors. After extensive tests on many types of mouse/human tissue, the accumulated total of spectrally resolved or spatially segmented (orthogonal) cellular and matrix molecular contrasts has surpassed that of detection channels/colors (Table II), highlighting the polymorphism of one spectral detection channel and the advantage of image segmentation in multimodal multiphoton microscopy (i.e., orthogonal content, Table I).

## IV. EMERGENT MULTICONTRAST LIVE-CELL IMAGING BY SLAM MICROSCOPY

To further extend the *in vivo* applicability, the aforementioned live-tissue imaging (Table II) should be adapted for live-cell imaging^38^ that emphasizes different samples, imaging geometries, extracellular matrices, and applications (Table III). The key motivation for this extension is to recognize and track the same types of unlabeled live cells, by an identifiable phenotype of cellular molecular contrasts from *in vivo*, intravital, or fresh *ex vivo* microenvironments, in an *in vitro* matrix amenable to human interventions.^40^ The practicability of using cultured cell lines and engineered matrix materials (hydrogel, matrigel, etc.) to model *in vivo* tissue can then be assessed by one common labelfree imaging tool. Thus, multiphoton microscopy can bridge and unify the live-tissue imaging dominated by laser-scanning confocal microscopy and the live-cell imaging dominated by a variety of wide-field microscopy (Table III), which have been technically isolated from each other.

**FIG. 5.**
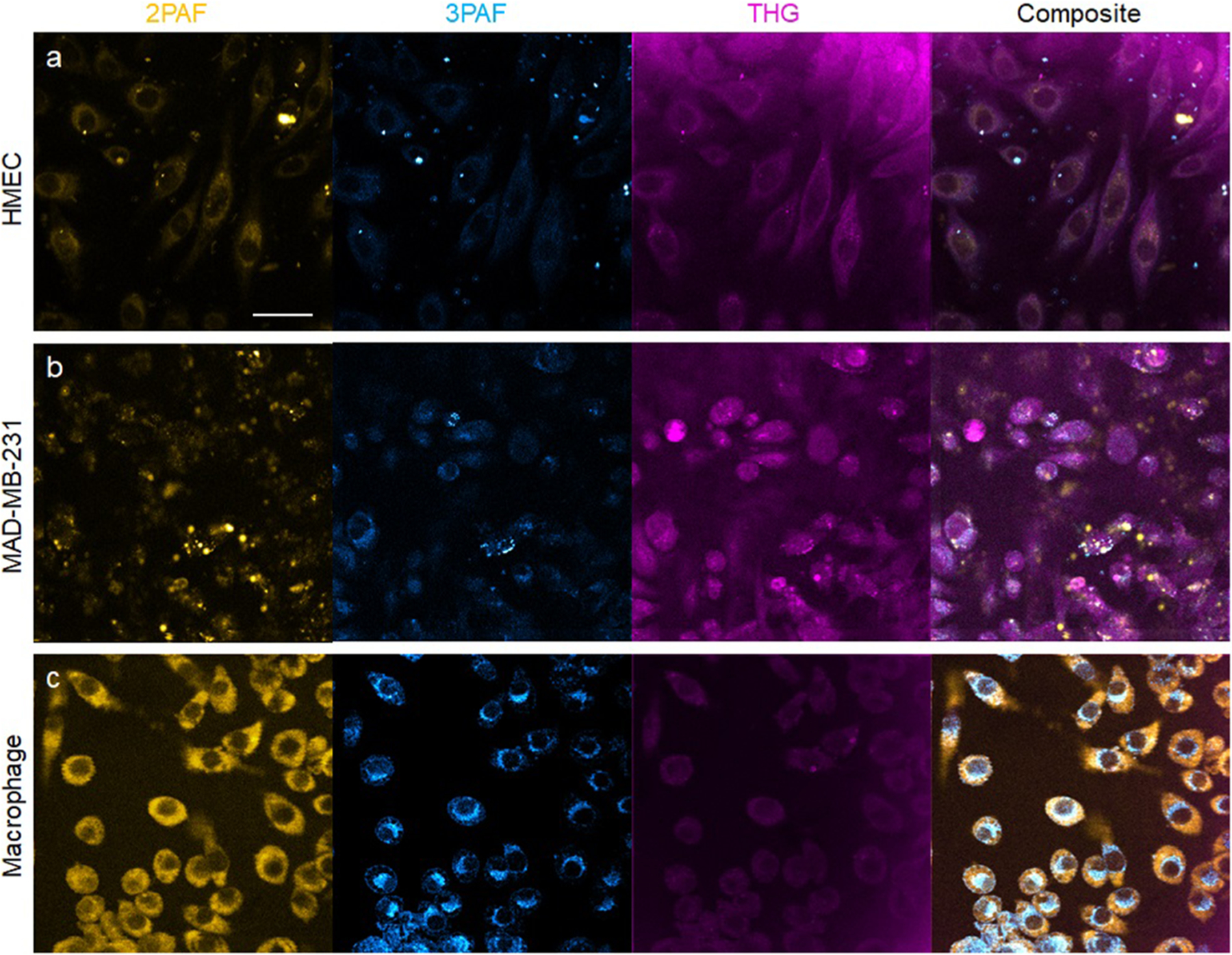
Live-cell imaging by SLAM microscopy. (a) Mammary epithelial cells (HMEC) cells, (b) breast cancer cells (MDA-MB-231), and (c) macrophages. Scale bar: 40 *μ*m (applicable to all fields of view).

With no modification to the optical geometry [Fig. 2(b)], cultured mammary epithelial cells (HMEC) and breast cancer cells (MDA-MB-231) can be imaged under the same condition (Fig. 5, Table II), except for 3-frame averaging over sequential raster scans to improve the SNR (i.e., effective pixel dwell time of ~30 *μ*s). Even though epicollected THG signal is weaker than forward-collected THG signal,^5^ the HMEC and MDA-MB-231 cells are easily identified by the THG channel, but not by the 2PAF or 3PAF channel [Figs. 5(a) and 5(b)]. On the other hand, cultured macrophages are easily identified by the 2PAF or 3PAF channel, but not by the THG channel [Fig. 5(c)]. These live-cell imaging experiments confirm the complementary nature of THG and autofluorescence signals observed in live-tissue imaging experiments [Fig. 4(e)], revealing the advantage of SLAM microscopy over its two precedents that employ either THG or autofluorescence (Table II). In contrast to the HMEC cells that show mitochondrial-like YRAFs/2PAF signal around the cell nucleus but punctuated cytoplasmic NADH/2PAF signal [Fig. 5(a)], the MDA-MB-231 cells exhibit mitochondrial-like NADH/2PAF signal but punctuated cytoplasmic YRAFs/2PAF signal [Fig. 5(b)]. Also, in contrast to the HMEC cells, the YRAFs/2PAF signal of the macrophages spreads from NADH/2PAF-highlighted mitochondrial-like regions to the whole cytoplasm [Fig. 5(c)]. These differences among normal epithelial cells, tumor cells, and immune cells can be *directly* evaluated in future live-tissue imaging.

It is often assumed that nonpunctuated cytoplasmic fluorescence throughout the visible spectrum (400–650 nm), which includes the 3PAF and 2PAF channels, arises from NADH and FAD exclusively.^2^ In other words, the laser source engineering not only targets cytoplasmic NADH via 3PAF but also cytoplasmic FAD via 2PAF. The 2PAF channel would collect FAD fluorescence at the red edge of its emission spectrum [Fig. 3(b), middle panel], which seems rather inefficient. However, it should be noted that the excitation wavelength of 1110 nm can only produce FAD/2PAF signal with a spectrally “blue-clipped” emission due to the energy difference between excitation and emission photons, in contrast to plausible simultaneously produced FAD/3PAF signal with a full emission spectrum [Fig. 3(b), middle and bottom panels]. To examine the nature of the observed FAD signal, an aqueous FAD solution can be prepared and then “imaged.” The dependence of the 2PAF channel intensity on the excitation power yields a photon order *n* of 2.2 (Table IV), indicating the dominance of the FAD/2PAF signal (*n* = 2) over FAD/3PAF signal (*n* = 3) [Fig. 3(b), middle panel]. Consistently, the latter generates only a small bleed through into the 3PAF channel (8%, Table IV). A similar experiment on an aqueous NADH solution yields *n* = 2.9 for 3PAF channel intensity, indicating the dominance of NADH/3PAF signal (*n* = 3) over NADH/2PAF signal (*n* = 2). In this case, the observed bleed through into the 2PAF channel (4%, Table IV) is attributed to a combination of the NADH/3PAF signal and plausibly the NADH/2PAF signal. While the prepared FAD and NADH solutions have an arbitrarily chosen concentration (100 *μ*M), this orthogonality between NADH and FAD signals can be theoretically justified to be independent of the concentration. The comparison of these solution experiments with real-time live-tissue imaging^10^ (pixel dwell time of ~10 *μ*s) indicates that 100 *μ*M of free NADH/FAD (lower concentration for protein-bound NADH due to increased quantum yield) is generally sufficient to reveal the mitochondria/cytoplasm of various cells via 3PAF/2PAF.

Thus, the laser source engineering creates an unusual condition to orthogonally collect single-shot NADH and FAD signals via regular spectral detection, which would be difficult if the two molecules are 2PAF-excited at ~800 nm to generate spectrally overlapped signals [Fig. 3(b), top panel]. For red-shifting excitation,^40^ the 1110-nm low-duty-cycle excitation is located at a “sweet” spot before the 2PAF-to-3PAF transition for FAD, but after the same transition for NADH. Lowering excitation duty-cycle *fτ* by 30-fold (Table II) is necessary to counter the decrease of FAD two-photon absorption cross section *σ*_2P_ from 900-nm to 1110-nm excitation [Fig. 3(b), bottom panel] so that the FAD/2PAF signal generation rate under the same average power [proportional to *σ*_2P_/(*fτ*)] may approach that excited at 900-nm and normal *fτ*.^2^ The simultaneous acquisition of NADH and FAD signals in one shot obviates the translation of the 2PAF-SHG platform from live-tissue imaging to live-cell imaging, which requires a tunable Ti:sapphire laser to collect the signals in two shots (Table II). This may simplify the measurement of the optical redox ratio to quantify cellular energy metabolism.^41^ It should be noted that the envisioned single-shot optical metabolic imaging requires that the cytoplasm-revealing 2PAF signal be indeed dominated by FAD fluorescence, just like the conventional two-shot (typically 740-nm and 900-nm, Table II) optical metabolic imaging.^2^ It remains unclear whether the modification of the excitation condition and the redshifted FAD detection (from 530 ± 30 nm to 610 ± 30 nm, Table II) would ease or worsen the contamination from non-FAD biomolecules. One source of contamination is lipofuscin that accumulates in lysosomes (not mitochondria), which can be spatially segmented from mitochondrial FAD within the same detection channel.^42^ For a similar reason, the mitochondrial-like and punctuated cytoplasmic YRAFs/2PAF signals, observed, respectively, from the HMEC cells and MDA-MB-231 cells [Figs. 5(a) and 5(b)], may be, respectively, attributed to FAD in the mitochondria and lipofuscin in the lysosomes.

In general, however, spectrally overlapped fluorescence from biomolecules are not amenable to this image segmentation. It is thus beneficial to introduce an additional red-shifted detection channel to selectively image non-FAD endogenous fluorophores such as porphyrin (650–800 nm, Fig. 3(b), middle panel).^43^ This channel will selectively reveal porphyrin-rich red blood cells (and other near-infrared fluorescent cells) that may be erroneously assigned as FAD-rich cells within one common channel of YRAFs/2PAF [Fig. 4(a), broken curves]. In the short wavelength end, it will be interesting to find out whether four-photon excited fluorescence^44^ of endogenous fluorophores (e.g., Trp) can be detected. To further differentiate biomolecules with spectrally overlapped fluorescence, the lifetime of autofluorescence can be simultaneously measured with its intensity. The lifetime is more amenable to quantitative analysis than the intensity due to its insensitivity to fluorophore concentration and excitation condition^45^ and may be useful to isolate reduced nicotinamide adenine dinucleotide phosphate (NADPH) in the NADH channel and non-FAD YRAFs in the YRAFs/FAD channel [Fig. 3(b), middle panel]. With acceptable temporal resolution, real-time collection of the lifetime information requires only fast electrical data acquisition, without modifying optics or decreasing imaging speed.^46^

The interaction between live-cell imaging and its live-tissue counterpart is bidirectional. Along the tissue-to-cell direction, prior live-tissue imaging will serve as the reference to assess how well the *in vitro* cell imaging approaches the *in vivo* condition in tissue. It is reasonable to assume that a “realistic” *in vitro* condition would reproduce both morphology and cellular molecular contrasts (Table II) for the cell type(s) of interest. Taking one step further, 3D collagen-rich hydrogels (rather than conventional 2D plastic/glass substrates or collagenfree 3D liquid suspensions) have been advocated to culture cells, spheroids, and organoids.^39,47^ The collagen signal in the hydrogels will be recovered in the SHG channel, which would otherwise be absent when imaging conventional (collagenfree) cell cultures (Fig. 5). It can be imagined that “realistic” hydrogel-assisted cell or spheroid/organoid cultures would not only recapitulate the *in vivo* dynamics of cells but also that of the collagen-revealed extracellular matrix. Along the opposite (cell-to-tissue, or translational) direction, *a priori in vivo* imaging of embryo development or the behavior of small organisms (*C. elegans*, zebrafish, fruit fly, etc.) may offer fundamental insights into whether similar processes can be observed in more complicated *in vivo* systems such as in mice or humans. The imaging studies across a continuum of spatial scales, temporal/developmental stages, and different species will translate the *in vivo* knowledge from simple multicellular organisms/embryos to humans. Overall, SLAM microscopy is expected to demonstrate significant benefits by seamlessly bridging between *in vivo* live-tissue imaging and *in vitro* live-cell imaging.

## V. RETHINKING TWO-PHOTON MICROSCOPY VIA DUAL-FLUOROPHORE BIOSENSING

In contrast to a standard or commercial two-photon microscope, a SLAM microscope gains general applicability in several aspects. First, the imaging is insensitive to ambient light contamination because its epi-imaging geometry allows simple light insulation for separated microscope and sample enclosures [Fig. 2(b)], regardless of whether it manifests as an inverted or upright microscope. In contrast, a forward-imaging geometry requires coupled microscope and sample enclosures and may hinder imaging under ambient light. Second, for the light-insulated sample enclosure, the imaging places no constraint on sample scale, origin, or nature (*in vitro*, *ex vivo*, *in vivo*, and intravital), regardless of whether the sample constitutes isolated (nanometer-sized) EVs from cell culture or large/extended microenvironments in living mice or humans,^25,26^ static embryos or dynamic small organisms, thick dissected or thin sectioned tissue, turbid (highly-scattering) or transparent (low-scattering) specimens, unlabeled/untreated or micromanipulated/photo-stimulated samples, etc. Third, the imaging is not restricted to a well-controlled environment to function only as benchtop instrumentation because its fixed-band excitation allows fiber laser source engineering to build robust portable instrumentation for diverse real-world situations beyond optical laboratories, such as for the intraoperative assessment of tumor margins.^26^ Just like optical coherence tomography (OCT) that employs similar fixed-band excitation, it is highly compatible with miniature biomedical imaging probes such as in endoscopy or handheld imaging devices to approach *in vivo* tissue in otherwise inaccessible locations [Fig. 2(b), right panel], and will potentially enable point-of-care *in situ* virtual biopsy.

**FIG. 6.**
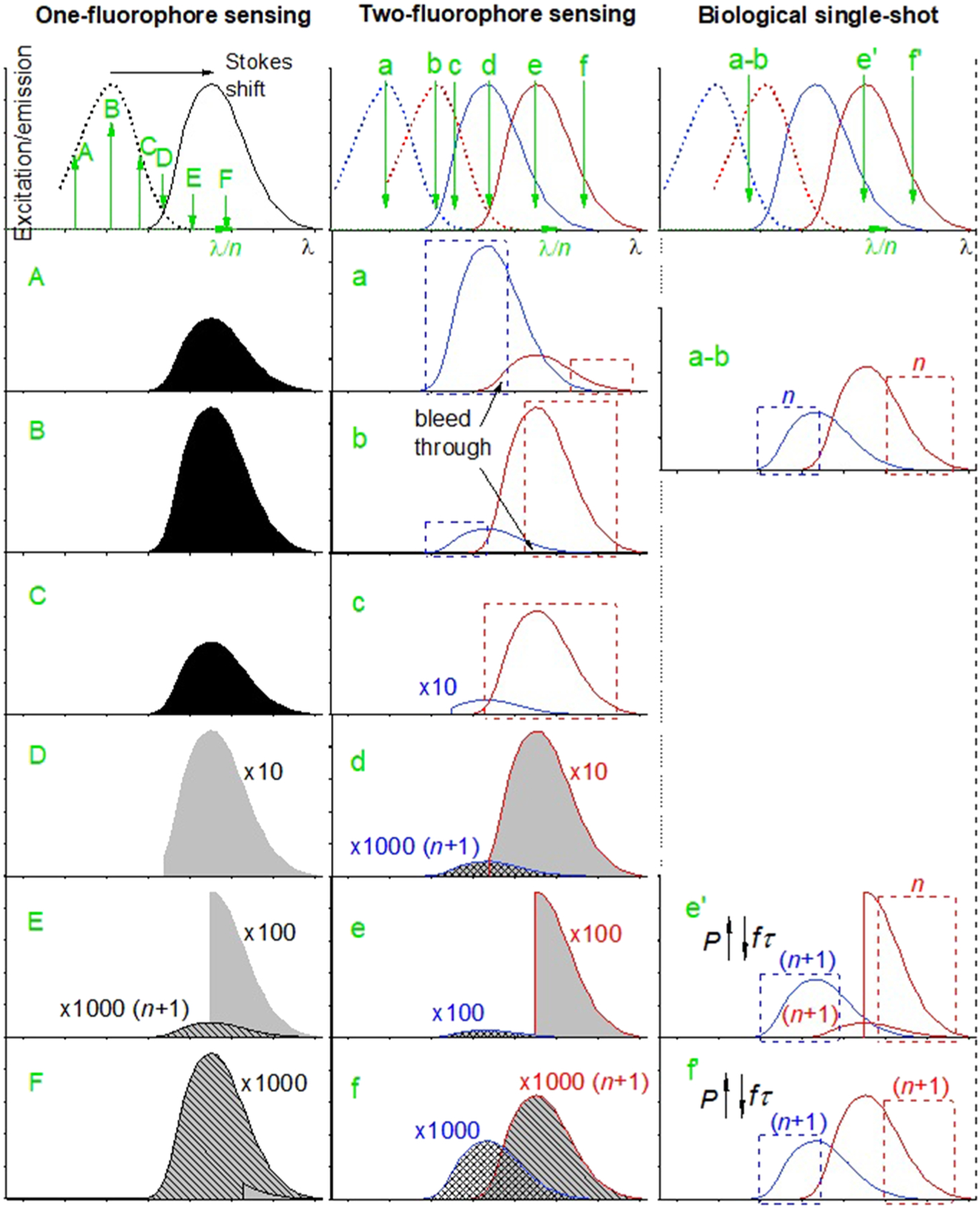
Mixed-photon-order processes and plausible blue-clipped fluorescence in one-fluorophore sensing (left panel), general dual-fluorophore sensing (middle panel), and biological single-shot dual-fluorophore sensing (right panel). For each panel, different subfigures reflect different extents of red-shift excitation.

A natural question arises as to what has caused these beneficial effects. In the following discussion, we will show that the root origin involves neither multiharmonic generation (SHG and THG) microscopy nor labelfree autofluorescence imaging, as the name implies. More surprisingly, it is not related to microscopy/imaging and biomolecule-targeted laser source engineering, even though they simplify the understanding of the underlying technology. It is indeed an underappreciated method to simultaneously sense in *in vivo* samples two fluorophores with spectrally overlapped fluorescence. Specifically, one relatively long-wavelength-emitting (red) fluorophore and another relatively short-wavelength-emitting (blue) fluorophore are sensed by a low photon-order (*n*) excited but blue-clipped fluorescence, and incrementally higher photon-order (*n* + 1) excited but full (nonclipped) fluorescence, respectively (Table IV, Fig. 6). Either the red fluorophore or the blue fluorophore, or both, may be of endogenous origin or be introduced exogenously. We will explain why such a highly specific feature in fluorescence sensing becomes the root cause for the above beneficial features and the favorable general applicability of SLAM microscopy.

A given fluorophore can be sensed via either linear/one-photon (*n* = 1) or nonlinear/multiphoton excitation (*n* > 1), and can be characterized by an *n*-photon-order excitation spectrum and an emission spectrum independent of *n* (known as Kasha’s rule) (Fig. 6, upper left).^48^ The linear/one-photon (*n* = 1) excitation spectrum and the emission spectrum can be measured by a commercial spectrofluorometer, while the *n*-photon-order (*n* > 1) excitation spectrum can be obtained by measuring the multiphoton absorption cross section.^44,49^ An overlap between the long wavelength end of the excitation spectrum and short wavelength end of the emission spectrum is often observed, reflecting the homogeneous broadening of the molecular transition in condensed matter physics and the Stokes shift associated with fluorescent emission (Fig. 6, upper left).^48^ To illustrate how a transition from low photon-order (*n*) excited fluorescence to incrementally higher photon-order (*n* + 1) excited fluorescence can occur, it is illuminating to excite this fluorophore with fixed average power *P* and duty-cycle *fτ* (for continuous wave excitation, *fτ* = 1) under a constant sensing (excitation and detection) condition, except for a red-shifting excitation wavelength *λ* (Fig. 6, left panel). In this way, the detected *λ*-dependent fluorescence intensity (integrated area over optical frequency range) follows the *n*-photon-order excitation spectrum [Figs. 6(A)–6(C)] until a point where significant blue-clipping is observed for the emission spectrum [Fig. 6(D)]. The blue clipping originates from the same principle that governs the Stokes shift, i.e., one emission photon of *n*-photon-order excited fluorescence has a lower energy than *n* excitation photons. Further red-shifting of *λ* will increase the blue-clipping and attenuate the *n*-photon-order excited fluorescence according to the excitation and emission spectra, until the emergence of (*n* + 1)-photon-order excited fluorescence [Fig. 6(E)]. Even further red-shifting will enable the dominance of the (*n* + 1)-photon-order excited fluorescence over its lower photon-order counterpart [Fig. 6(F)], completing the transition from low photon-order (*n*) excited fluorescence to incrementally higher photon-order (*n* + 1) excited fluorescence.

The above one-fluorophore sensing can be extended to the two-fluorophore sensing with spectrally overlapped excitation and emission spectra (Fig. 6, middle panel). At a short *λ*, the *n*-photon-order excited fluorescence of the blue fluorophore is much stronger than that of the red fluorophore so that a spectral window can be implemented to selectively sense the blue fluorophore with minimum bleed through [Fig. 6(a)]. A similar condition to selectively sense the red fluorophore can be attained by a proper redshift in *λ* [Fig. 6(b)]. An intermediate condition can be attained for balanced detection of both fluorophores with one shot [Figs. 6(a) and 6(b)]. Further red-shifting in *λ* will produce an attenuated blue-clipped fluorescence for the blue fluorophore so that a larger spectral window can be used to selectively sense the red fluorophore [Fig. 6(c)]. Along the direction of further *λ* red-shifting, the transition from low photon-order (*n*) excited fluorescence to incrementally higher photon-order (*n* + 1) excited fluorescence happens earlier for the blue fluorophore than the red fluorophore [Figs. 6(d)–6(f)], resulting in an interesting condition that the *n*-photon-order excited blue-clipped fluorescence of the red fluorophore dominates its counterpart of the blue fluorophore while the (*n* + 1)-photon-order excited full fluorescence of the blue fluorophore dominates its counterpart of the red fluorophore [Fig. 6(e)]. Although this one- and two-fluorophore sensing (Fig. 6, left and middle panels) represents an oversimplified picture for general fluorescence sensing, the qualitative features of this picture remain valid for highly different excitation and emission spectra.

If we would like to avoid optical sources with a higher peak intensity (higher *P* and/or lower *fτ*), which often incur a higher cost, the relatively weak blue-clipped or higher photon-order excited fluorescence signals excited at longer *λ* cannot be increased by elevating the peak intensity. Thus, there is little incentive to use multiphoton excitation in nonbiological fluorescence sensing because the same signal can often be detected by single-photon excitation more efficiently and economically. Consequently, two-photon fluorescence sensing has only found limited applications in microvolume bioassays, capillary electrophoresis, microfluidics, and chromatography, in which one-photon fluorescence sensing would be hindered by small sample volumes or expensive optics or substrates for transmitting deep ultraviolet (UV) signals.^50–52^ However, the situation changes in *in vivo* fluorescence sensing that favors single-shot two-fluorophore sensing with low photodamage risks because the biological sample is more tolerable to the high peak intensity (high *P* and/or low *fτ*) at longer *λ*. Since the *n*-photon-order excited fluorescence signal scales with *P*^*n*^/(*fτ*)^*n*−1^, comparable signals can be obtained from *n*- or (*n* + 1)-photon-order excited (blue-clipped) fluorescence at longer *λ* [Figs. 6(e) and 6(f)] and *n*-photon-order excited fluorescence at shorter *λ* [Figs. 6(a) and 6(b)]. In the case of *n* = 1, multiphoton excitation [Figs. 6(e) and 6(f)] often achieves a higher signal-to-photodamage (or signal-to-photobleach) ratio than single-photon excitation [Figs. 6(a) and 6(b)].^53^ More importantly, the mixed-photon-order two-fluorophore sensing that invokes the blue-clipped fluorescence of the red fluorophore [Fig. 6(e)] can detect the signals more efficiently (reject less signals to avoid bleed through) than the uniform photon-order two-fluorophore sensing of full fluorescence [Figs. 6(a), 6(b), and 6(f)] either in linear (*n* = 1) or nonlinear (*n* > 1) optical regime. This blue-clipped fluorescence has been invoked to limit its bleed through (background contamination) into the intended signal [comparing Figs. 6(c) and 6(b)], but has rarely been pursued as the signal itself due to the common perception of its inefficient signal generation at the red edge of excitation spectrum [Fig. 6(e)].

One specific case of the mixed-photon-order two-fluorophore biosensing with blue fluorescence clipping to the red fluorophore, termed clipping-assisted dual-fluorescence sensing (CADFS), employs mixed two- and three-photon excitation [*n* = 2 in Fig. 6(e’)] of FAD and NADH, arguably the two most important endogenous fluorophores [Fig. 3(b)]. The excitation wavelength (1110 nm) generates a highly blue-clipped FAD fluorescence so that the otherwise strongly overlapped emission spectra of NADH and FAD becomes relatively well-resolved for orthogonal spectral detection [Fig. 3(b), middle panel]. For simplicity, SLAM microscopy is treated as a specific application of dual-fluorophore sensing, even though it may be more appropriate to attribute the blue fluorophore to NADH-like endogenous fluorophores including NADPH, and fatty acids, and the red fluorophore to FAD-like endogenous fluorophores including lipofuscin and flavin mononucleotide. Similarly, other variants of this generalized CADFS can be implemented to detect different pairs of blue and red endogenous fluorophores, leading to different imaging conditions with distinct advantages and limitations (Table V).

Although these variants can be easily differentiated by their distinct excitation wavelengths, the access to them does not guarantee a high performance. For example, photodamagefree biosensing of NADH and FAD via 3PAF (or porphyrin via blue-clipped 2PAF) at ~1200 nm excitation is not possible by the use of a compact Cr:forsterite laser (*fτ* = 10^−5^) (Table II), but possible by the use of a bulky optical parametric amplifier (*fτ* < 10^−6^) (Table V). The decreased *fτ* is necessary to limit the average excitation power *P* on sample and the corresponding thermal photodamage. This is why all these variants demand a *fτ* that is at least one order-of-magnitude lower than that of typical commercial femtosecond lasers (Table V). The lack of readily available low-*fτ* lasers may have been the cause for overlooking CADFS in well-established labelfree multiphoton imaging.^1–3^ In fact, mixed-photon-order two-fluorophore biosensing of NADH and Trp has been realized by a regular high-*fτ* Ti:sapphire laser at<740 nm excitation (Table II), with no blue clipping to the NADH fluorescence.^3,28^ Plausibly due to a lack of low-*fτ* Ti:sapphire lasers, the excitation has not been shifted to 800 nm to obtained a balanced sensing of NADH (blue-clipped 2PAF) and FAD (full 2PAF) [Figs. 6(a) and 6(b)], as well as Trp (full 3PAF) (Table V). The blue clipping to the otherwise strong NADH/2PAF fluorescence (shifting *λ* to 800 nm) is required to expose the weak FAD/2PAF fluorescence, in contrast to the case of 1110 nm excitation where the blue clipping to the otherwise strong FAD/2PAF fluorescence is required to expose the weak NADH/3PAF fluorescence. Without the blue clipping, the corresponding full fluorescence will obscure a weak but orthogonal (informative) fluorescence and forbid the balanced sensing of two responsible fluorophores. Also, the redshift in excitation wavelength that accompanies the blue clipping has enabled simultaneous THG and SHG epidetection, defying the common perception that THG imaging is incompatible with fluorescent imaging of NADH and FAD under one common excitation. The synergistically integrated labelfree fluorescence and harmonic imaging has subsequently led to the aforementioned beneficial features and favorable general applicability of SLAM microscopy.

The CADFS technology is rather unintuitive because it must intentionally clip a fluorescence signal of interest, and as a result, attenuates this signal that could otherwise be detected with higher (>10-fold) efficiencies. However, this cost is justified by gaining another fluorescence signal that would be obscured by the fluorescence signal without attenuation, enabling a simple shift from multishot to single-shot two-fluorophore sensing. With the recognition of CADFS as the core technology for SLAM microscopy, it is now possible to rethink the conventional implementation of two-photon microscopy and the related nonimaging applications based on tunable (700–1050 nm) Ti:sapphire lasers or more widely tunable (700–1300 nm) optical parametric oscillators, but with a fixed and relatively high *fτ* (~10^−5^). This conventional implementation will be challenged by CADFS in several aspects. First, CADFS is not limited to two-photon processes. It down-shifts *fτ* of a nontunable laser to enable three-photon microcopy and balanced detection of two- and three-photon excited signals, and thus attains favorable general or *in vivo* applicability through the one-shot strategy. Second, unlike SLAM microscopy, CADFS is not limited to labelfree imaging and will empower conventional two-photon fluorescence microscopy to simultaneously detect two fluorophores with highly spectrally overlapped fluorescence. The two fluorophores may originate from either autofluorescence or exogenous labeling, or a combination of the two, offering numerous opportunities to enhance the biological imaging of interest. Third, CADFS is not limited to imaging and can be used to enhance the nonimaging applications of two-photon excitation, such as *in vivo* flow cytometry where the sensing occurs in the animal (or potentially human) body and *in vitro* flow cytometry when viable cells must be harvested after cell sorting.^54,55^

## VI. CONCLUDING REMARKS AND FUTURE DIRECTIONS

In summary, the beneficial features of the single-shot multimodal multiphoton microscopy include: (1) labelfree imaging of multiple molecular contrasts in one shot, with comprehensive structural, functional/molecular, and metabolic information for both cells and extracellular matrices; (2) favorable *in vivo* applicability enabled by a specific configuration vector for optical excitation and signal detection, in a widely available form of commercial laser-scanning confocal microscopy; (3) simple fixed-band laser source that resembles the widely used source for OCT structural imaging, but intrinsically targets ubiquitous biomolecules of collagen, lipid, NADH, and to a less degree, FAD, by multimodal multiphoton imaging modalities along different spectral detection channels; (4) ability to perform optical molecular profiling independent of the spatial scale of biological structures (from submicron vesicles to large-scale vessels); (5) orthogonal image segmentation that allows collecting more molecular contrasts than the number of imaging modalities or spectral detection channels; (6) seamless integration of often isolated live-tissue imaging and live-cell imaging by one optimized excitation condition, with a tight window of excitation parameters to balance diverse trade-offs; and (7) favorable general applicability for biophotonic imaging with minimum training for nonexperts, and minimum constraints on the sample, imaging geometry, miniaturization, working environment, and biological or biomedical subfield.

Our discussion so far is more relevant to *applied* research than *basic* research. From the perspective of applied research, the emergence of CADFS technology and subsequent SLAM microscopy has defied the conventional wisdom that high flexibility in the laser source (e.g., tunable wavelength) is necessary for multiphoton imaging/sensing with favorable general applicability. We envision that future laser source engineering for multiphoton microscopy will diverge from the tunable Ti:sapphire lasers (workhorses) along two directions. In one direction relevant to the basic research and the multishot strategy, the lasers will be designed to achieve not only a wide range of tunable wavelengths but also a high degree of flexibility in duty-cycle, preferably in a programmable user-friendly platform. This platform will be useful to identify discrete optimal conditions of the excitation wavelength and duty-cycle for specific biomolecules of interest. In fact, the excitation condition that has first enabled SLAM microscopy was derived from a programmable fiber supercontinuum source that embraces the multishot strategy.^9^ For a wide variety of live-cell and live-tissue samples, we have found that this excitation condition has improved the signal-to-photodamage ratio over that from typical tunable Ti:sapphire lasers. It is possible that other optimal conditions employing a different CADFS technology [*n* ≠ 2 in Fig. 6(e)] may find different applications or mitigate photodamage. In the other direction relevant to the applied research and the single-shot strategy, the lasers will be designed to emit constantduty-cycle fixed-wavelength pulses at the identified optimal conditions that target specific biomolecules, preferably in a compact fiber laser platform suitable for robust portable applications (e.g., Table V, 1035 nm).^56^

There are several promising directions for further improvement. First, the excitation parameters (1110 nm, 30 fs, 10 MHz, 14 mW) may be further optimized for general biological imaging. A longer wavelength will improve THG imaging,^5^ but would attenuate the blue-clipped FAD/2PAF signal. A shorter pulse will generate larger signals, but would decrease spectral selectivity in multimodal spectral detection under broader band excitation.^11^ A lower repetition rate will generate larger signals and improve deep-tissue imaging, but would slow down the speed in laser-scanned imaging where at least one pulse per pixel is required for signal acquisition. A higher average power will enable faster imaging, but would increase the risk of thermally mediated photodamage. To avoid different mechanisms of photodamages, the selections of laser parameters (wavelength, pulses width, repletion rate, and average power) have been systematically studied recently in mouse brain imaging.^57^ These trade-offs impose a tight window for the laser parameters. Second, additional autofluorescence detection channels and contrasts (i.e., lifetimes of all autofluorescence detection channels) may be introduced to gain more information [Fig. 3(b), middle panel], while novel photo-detectors (e.g., HyD hybrid detector, Leica Microsystems) can be used to measure both fluorescence intensity and lifetime in real time. Also, imaging contrasts can be enhanced by methods such as fluorescence recovery after photobleaching and polarization-sensitive harmonic generation. Third, software-based deconvolution and hardware-based adaptive optics may be implemented to improve the image quality and deep-tissue volumetric imaging, respectively. Fourth, the demonstrated laser-scanning imaging may be adapted for light-sheet or wide-field excitation and planar signal detection (e.g., CCD or CMOS camera) to speed up volumetric imaging, paralleling the similar adaptations of multiphoton and confocal microscopy.^58,59^

One limitation of SLAM microscopy is the relatively low resolution (~0.5 *μ*m) at 1110-nm excitation. The saturated fluorescence imaging by wide-field structured illumination may improve the resolution of the 2PAF/3PAF signal^60^ but will not improve that of the SHG/THG signal. A uniform resolution improvement across all imaging modalities may employ a dark beam imaging method known as Switching LAser Modes microscopy, which serendipitously has the same abbreviation (SLAM).^61^ The perspective switching-laser-modes SLAM microscopy (or SLAM-squared microscopy) will gain higher spatial resolution at the cost of two-shot excitation, which will be valuable for the relatively static live-cell imaging to better resolve subcellular organelles (e.g., mitochondria and lysosome). Another limitation is the rather small field-of-view (0.3 × 0.3 mm^2^) typical for laser scanning confocal/multiphoton microscopy. This limitation can be mitigated by stitching a 2D mosaic matrix of multiple fields-of-view after mechanical stage scanning. A better solution is to integrate an OCT module using the same broadband excitation source but employing switchable objectives that alternate between large-volume low-resolution imaging (OCT) and small-volume high-resolution imaging (SLAM). This will be useful to quickly scan a large specimen and zoom-in smaller regions of interest.

Historically, the coupling of a Kerr-lens mode-locked wavelength widely tunable Ti:sapphire laser with two-photon microscopy in 1992 integrated the “magic” of Kerr-lens mode-locking developed by laser physicists with the “magic” of scanned nonlinear optical illumination developed by bioimaging scientists and has thus formed the conventional biological two-photon microscopy.^62^ However, a quarter century later, this conventional two-photon microscopy and related nonimaging applications remain largely restrictive to a small number of optical laboratories or biological laboratories with engineering expertise and is operated more often by laser experts than by biologists. It is our hope to empathize an alternative laser source engineering toward a fixed but “smart” choice of parameters (wavelength, bandwidth, pulse width, repetition rate, etc.), rather than increased flexibility (complexity) for these parameters that causes user unfriendliness, environmental instability (immobility), and high cost to limit biological nonlinear optical microscopy. It is conceivable that a series of popular, if somewhat oversimplified, cellular-extracellular components can be molecularly segmented with information content maximized at increasingly optimized excitation and detection conditions. We envision that the resulting multiphoton imaging will rival or outperform alternative and more popular methods for biological/biomedical optical imaging, will help researchers to recreate complex *in vivo* microenvironments *in vitro* and perform fast point-of-care diagnosis of diseases, and will ultimately impact both bioscience and medicine.

## Figures and Tables

**TABLE I.** Survey of representative platforms of labelfree molecular nonlinear optical microscopy.

	SRS molecular^13^	CARS spectroscopic^14^	Single-shot multimodal multiphoton^3,10^	Single-beam integrated^15^	Adaptive multishot^9^
Modalities included	SRS	CARS	Combinations of 2PAF, 3PAF, SHG, THG	2PAF, CARS, SHG, THG	2PAF, 3PAF, CARS, SHG, THG
Laser source	Two synchronized ps-fs lasers	Custom-built ps-fs continuum source	Commercial or custom-built lasers	Ultrabroadband Ti:Sa laser	Custom-built programmable continuum laser
Single-shot (5)	−(0)	+(5)	+(5)	±(2.5)^δ^	−(0)
CRS inclusion (4)	+(4)	+(4)	−(0)	+(4)	+(4)
Orthogonal content (3)	±(1.5)^α^	±(1.5)^α^	+(3)	+(3)	−(0)
Epidetection (2)	±(1)^β^	−(0)	+(2)	−(0)	+(2)
Single-beam (1)	−(0)	−(0)	+(1)	+(1)	±(0.5)^ε^
Total applicability score	6.5	10.5	11	10.5	6.5
Limiting factor	Precise spectral calibration	Slow speed hindering real-time imaging	Plausible lack of orthogonal molecular contrast	Special UV optics for THG, lack of orthogonal contrast	Highly customized broadband laser

Note:

α- spectral detection or excitation without multimodal multiphoton modalities;

β- not ideal but possible;

δ- applicable to multiphoton modalities but not CARS;

ε- applicable to multiphoton modalities but not CARS.

**TABLE II.** Representative platforms for labelfree single-shot multimodal multiphoton microscopy.

Platform	2PAF-SHG(−3PAF)^3,28^	THG-SHG(−2PAF/3PAF)^23,27^	SLAM^10^
Laser source	Commercial (tunable) Ti:sapphire	Custom-built Cr:forsterite or commercial tunable OPO	Custom-built filtered supercontinuum
Pulse width *τ* (fs)	150	150	30
Repetition rate *f* (MHz)	80	~80	10
Duty-cycle *fτ*	1.2 × 10^−5^	1.2 × 10^−5^	3.5 × 10^−7^
Excitation wavelength (nm)	740	1230	1110
Average power *P* on sample (mW)	<20	<100	14
Fluorescence detection color	360 ± 20 nm (3PAF), 460 ± 30 nm (2PAF)	>630 nm (2PAF)	450 ± 30 nm (3PAF), 620 ± 30 nm (2PAF)
Channel/color/detector	2 or 3	2 or 3	4
Cellular molecular contrast	NADH/2PAF, (Trp/3PAF)^α^	Membrane-lipid/THG	NADH/3paf, membrane-lipid/thg, YRAFs/2PAF
Matrix molecular contrast (matrix refers to the stroma surrounding epithelial cells)	Collagen/SHG, myosin/SHG, lipid-vacuole/2PAF, elastin/2PAF	Collagen/SHG, myosin/SHG, myelin/THG, EV/THG, lipid-vacuole/THG, elastin/(THG)	Collagen/SHG, myosin/SHG, myelin/THG, EV/THG, lipid-vacuole/(THG, 3PAF), elastin/2PAF, elastinlike/THG
Distinct strength	Metabolic function	Extracellular matrix structure	All of the left
Key limitation	Missing lipid (backup of cellular and extracellular contrast)	Missing NADH (function, metabolism)	None of the left
Often required adaption from live-tissue to live-cell imaging	Tune to ~900 nm to detect FAD/2PAF (650 ± 30 nm, typically)	Detect transmitted rather than reflected (epi) signal	None of the left

Note:

α- may not be counted as an independent contrast because related SHG emission locates near the center of Trp emission spectra [Fig. 3(b), top panel] so that it is technically challenging to simultaneously collect both SHG and likely weak Trp/3PAF signals using regular dichroic mirrors and bandpass filters.

**TABLE III.** Comparison of two distinct categories of multiphoton microscopy.

	Live-tissue multiphoton microscopy	Live-cell multiphoton microscopy
Dominant alternative tissue/cell imaging	Laser-scanning confocal microscopy with single-element (PMT) detector(s)	Wide-field transmission/fluorescence microscopy or light-sheet microscopy with planar (CCD) detector(s)
Sample nature	Thick/large samples with high scattering	Thin or clear samples with low scattering
Sample survey	Skin, internal organs accessible by chronic imaging window (mouse) and endoscopy (human), small tissue biopsies, large surgical specimens, exposed cavity during surgery	Cultured cells, spheroids, and organoids, developing embryos, model multicellular organisms such as *C. elegans*, zebrafish, and fruit fly, thin tissue of mouse ear, mesentery, or cremaster muscle in an intravital setting
Sample condition	Intravital (mouse tissue in general), fresh *ex vivo* (animal/human), and *in vivo* (human)	*In vitro* (cells, spheroids, organoids), *in vivo* (embryos, small organisms), intravital (thin mouse tissue)
Preferred imaging method/geometry	Fast (real-time), single-shot, labelfree, and epidetected imaging	More molecular contrasts with less need on single-shot labelfree imaging and signal epidetection
Signal detection geometry during imaging	Epidetection possible due to signal backscattering, transmitted signal detection impossible due to thick sample obstruction	Episignal detection impossible for SHG without backscattering, transmitted signal detection possible and preferred for SHG and THG
Continuous (not snapshot) imaging	Longitudinal intravital study using chronic imaging windows	Time-lapse microscopy in an incubatorlike environment
Extracellular matrix	In authentic tissue microenvironment	Often in culture media amenable to human interventions
Main applications	Pathology, (pre-)clinical disease monitoring	Therapy, drug development, tissue engineering
Related applications	Radiology, endoscopy, surgical oncology	Cytometry, microfluidics, biopharmaceutical industry

**TABLE IV.** Validation of FAD/2PAF and NADH/3PAF signals and their orthogonality.

	2PAF (610 ± 30 nm)	3PAF (450 ± 30 nm)	Order of nonlinear optical process
FAD solution (100 *μ*M)	100% (14 mW)	8% ± 2% (14 mW)	*n* = 2.2 ± 0.1 (2PAF channel, 2–14 mW excitation)
NADH solution (100 *μ*M)	4% ± 1% (14 mW)	61% (14 mW)	*n* = 2.9 ± 0.1 (3PAF channel, 2–14 mW excitation)

**TABLE V.** Variants of CADFS technology for fluorescence sensing.

Targeted fluorophore pair	Trp-(NADH/FAD)	NADH-FAD		(NADH/FAD)-porphyrin
Blue-clipped 2PAF signal	NADH	FAD		Porphyrin
Full 3PAF signal	Trp	NADH		NADH/FAD
Excitation wavelength *λ* (nm)	800	1035 nm	1110	~1200
Duty-cycle *fτ*		<10^−6^		
Commercial or custom laser source (size)	Commercial Ti:sapphire amplifier (bulky)	Commercial Yb fiber amplifier (compact)	Custom source (intermediate)	Commercial optical parametric amplifier (bulky)
Advantage(s)	Information of Trp-NADH förster resonance energy, quantitative redox NADH-FAD metabolic	Plausible single-shot NADH-FAD-porphyrin imaging by fiber laser source	Plausible single-shot NADH-FAD-porphyrin imaging	Quantitative ratiometric NADH-FAD-lipid/THG live-cell imaging, deep-tissue imaging
Limitation(s)	Imaging Impaired THG for live-tissue imaging, photodamage due to short excitation wavelength	Impaired THG and quantitative redox NADH-FAD metabolic imaging	Impaired quantitative redox NADH-FAD metabolic imaging	Weak autofluorescence signals in comparison to harmonics signals
